# Varicella-Zoster Virus Reactivation in AIDS Patient After Pfizer-BioNTech COVID-19 Vaccine

**DOI:** 10.7759/cureus.20145

**Published:** 2021-12-03

**Authors:** Raed Atiyat, Samir Elias, Chrystina Kiwan, Hamid S Shaaban, Jihad Slim

**Affiliations:** 1 Internal Medicine, Saint Michael's Medical Center, Newark, USA; 2 Medical Education, Saint George University, True Blue, GRD; 3 Hematology and Oncology, Saint Michael's Medical Center, Newark, USA; 4 Infectious Diseases, Saint Michael's Medical Center, Newark, USA

**Keywords:** immunocompromised, aids, adverse effect, vaccination, covid

## Abstract

Since early 2020, severe acute respiratory syndrome coronavirus 2 (SARS-CoV-2) has affected millions of individuals and changed the face of medicine. As the fight against COVID continues, there is still unclear long term effects; although as time passes, more and more is being updated, in regards to the risks of exposure, length of recovery, outcomes of those infected, effectiveness of vaccines, and both expected and unique side effects of both the virus and vaccines, all in an array of individuals. This paper will review a unique topic of the SARS-CoV-2 virus and the abnormal immune response in a young patient. This case is unique due to the fact that there have been an abundance of side effects reported that are associated with the virus that affects every organ system, yet very few have affected the neurological and integumentary (skin) system. This case emphasizes the reactivation of a Herpes/Varicella-Zoster virus (VZV) in a young male shortly after he received the Pfizer-BioNTech COVID-19 vaccine. The other interesting aspect about this case is the patient’s immunocompromised state, as he was diagnosed with HIV several years before this viral reactivation occurred. The interesting aspect about this was trying to understand whether the VZV was truly reactivated because of an overly stressful immune reaction in response to the Pfizer-BioNTech COVID-19 vaccine or was it mainly due to the patient’s already weak immune system, or even a combination of both? The in-depth review will evaluate whether there should be more done in regards to bringing more awareness about potential side effects and preparing for a VZV reactivation and/or other dermatological complications after being vaccinated. This presentation could also simply be a very unique, isolated case, and that each individual should have no hesitations regarding the Pfizer-BioNTech COVID-19 vaccine.

## Introduction

Severe acute respiratory syndrome coronavirus 2 (SARS-CoV-2) is a positive-stranded RNA (+ssRNA) virus with a crown-like appearance under an electron microscope due to the presence of spike glycoproteins on the envelope. SARS-CoV-2 causes ACE/ACE2 balance disruption and RAAS activation, which leads ultimately to COVID-19 disease progression, especially in patients with comorbidities, such as hypertension, diabetes mellitus, and cardiovascular disease. Therefore, ACE2 expression may have paradoxical effects, aiding SARS-CoV-2 pathogenicity, yet conversely limiting viral infection.

Varicella-Zoster virus (VZV) is a human herpesvirus that primarily causes chickenpox (varicella) that can activate and reactivate at various stages throughout one’s life [1]. Chickenpox primarily occurs in children and is characterized by a generalized, pruritic, vesicular rash. After the childhood rash clears, the VZV virus remains latent in neural tissues and can reactivate many years later and at any point in the future [1]. The reactivation of the dormant VZV also known as Zoster (Shingles) is characterized by a localized maculopapular rash typically unilateral that is confined to one or two adjacent dermatomes. Reactivation is more commonly seen in the elderly population, usually in patients age 60 or above secondary to the waning cell-mediated immunity that occurs with aging in addition to other causes leading to a compromised immune system [2,3]. The initial signs of shingles include paresthesia, followed by rash consisting of blisters that usually crust, scab and heal in the coming 7-10 days and resolve in the coming month. Unfortunately, roughly 20% of patients suffer from post-herpetic neuralgia which can last from months to years [4].

Since the implementation of the COVID-19 vaccinations, there have been adverse effects ranging from fever, myalgia, Bell’s palsy, myocarditis, cutaneous manifestations such as urticaria, herpes reactivation, morbilliform rashes, maculopapular eruptions, and livedo reticularis. Below we discuss the first reported case of VZV reactivation in an AIDS patient after receiving COVID vaccine.

## Case presentation

A 36-year-old male presented to the emergency department with a unilateral ventral portion of right arm and right anterior chest painful rash that had been present for one week. The patient stated that after he received the second dose of the Pfizer COVID-19 vaccine in his left deltoid, he started to have a burning sensation all throughout his right upper extremity and midsternal area the next day. Two days later, the patient started developing blisters and vesicles, followed by the development of a red, maculopapular rash that was tender upon palpation, covering the patient’s ventral portion of right arm, forearm, and right midsternal chest area. He attempted multiple self-remedies, such as ibuprofen, aspirin, hydrocortisone lotion, Icy-Hot topical pain reliever, calamine spray, all within the course of one week, with no success. Furthermore, the patient stated that he consumed 1.75 L of vodka daily to try and alleviate the pain, with no success. Patient could not bear the pain and decided to visit the emergency department. 

**Figure 1:**
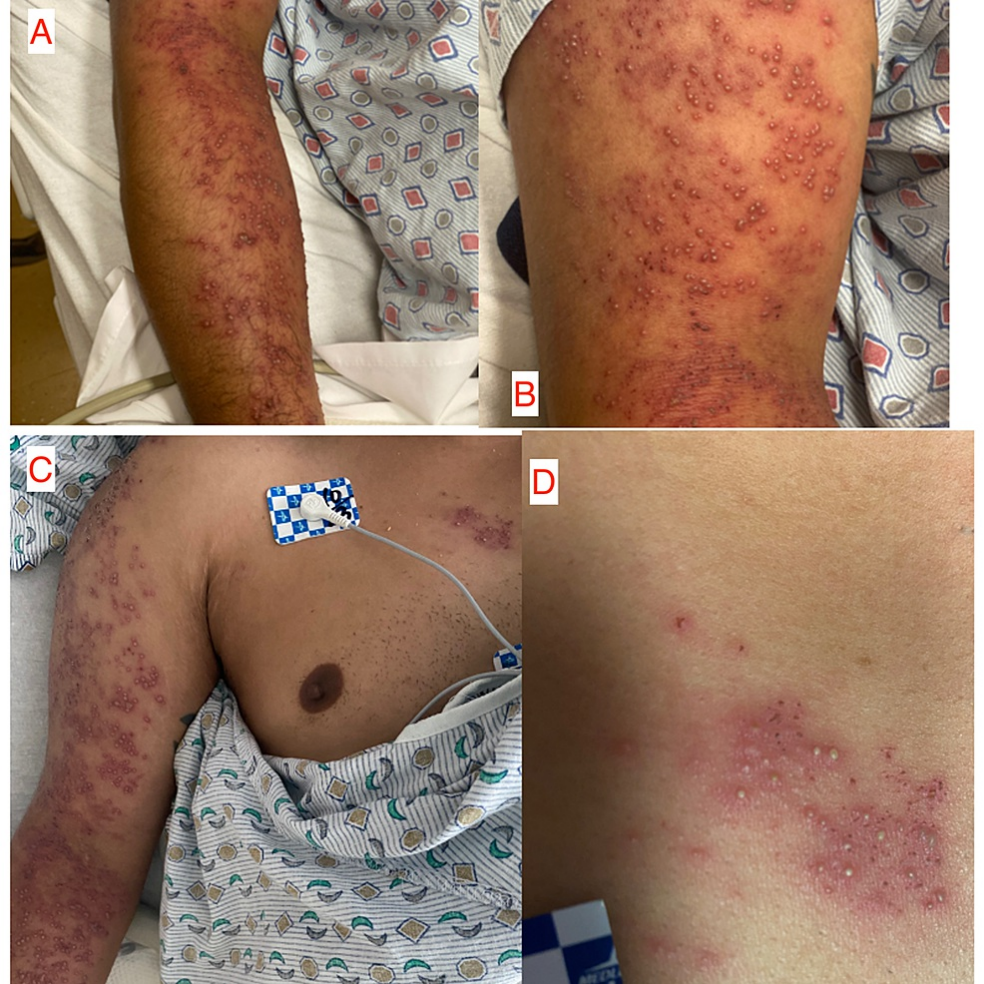
Rash on the ventral portion of right arm extending from the deltoid to his wrist appreciated in plane A and B . Right anterior chest around the T2-T3 area consisted of an erythematous rash with many vesicles appreciated in plane C and D.

Vitals on admission were a temperature of 99 °F (37.2 °C), a blood pressure of 147/106, a pulse of 120, a respiratory rate of 18, and an SpO2 of 98 % on room air. On the physical exam, the patient’s rash on the ventral portion of right arm extending from the deltoid to his wrist is seen in Figure 1, planes A and B. On his right anterior chest around the T2-T3 area consisted of an erythematous rash with many vesicles as appreciated in Figure 1, planes C and D. As per the patient, this rash was similar to the one he had developed previously on the left lower quadrant (LLQ) of his abdomen three to four years ago, when he had been diagnosed and treated for shingles. The patient otherwise was awake, alert and oriented to time, place and person but was very anxious and was tremulous when asked to stretch out his arms. The remainder of the physical exam was non-contributory. He also denied experiencing any headaches, blurry vision, chest pain, dyspnea, palpitations, abdominal pain, nausea, vomiting, diarrhea, constipation, urinary changes or weakness in his extremities. Patient had also denied any jerking motion, tongue biting, loss of consciousness, or urinary or bowel incontinence. Upon further questioning, the patient stated that he currently has HIV which was diagnosed in seven years prior, but was never compliant with his antiretrovirals. 

Laboratory results (complete blood count and comprehensive metabolic panel) on presentation were relatively unremarkable. Mild elevation in AST 45 U/L (10 - 36 U/L) and ALT 54 U/L (9 - 46 U/L), respectively. SARS-COV-2 PCR and antigen tests were negative. 

The following labs were done in regards to his HIV status seen below in Table 1 and Table 2. 

**Table 1:** HIV status.

HIV status	
CD4 count	158 (normal 359-1519)
HIV-1 RNA via PCR	20,600 copies/mL
HIV 1 Ab	Reactive
HIV 2Ab	Non-Reactive
HIV Ab Interpretation	HIV 1 Positive
Hep C Ab	Non-Reactive
Hep B surface Ag	Non-Reactive
Hep B surface Ab	Non-Reactive
RPR / Syphilis	Non-Reactive
Neisseria Gonorrhoeae	Non-Reactive

**Table 2:** Lymphocyte T cell panel.

Lymphocyte T cell panel	
Absolute CD 3	1846 (reference: 622-2402/uL)
Absolute CD 4 Helper	158 (reference: 359-1519/uL)
Abs. CD 8 Suppressor	1650 (reference: 109-897/uL)
% CD 3 Pos. Lymph.	83.9 (reference: 57.5-86.2%)
% CD 4 Pos. Lymph.	7.2 (reference: 30.8-58.5%)
% CD 8 Pos. Lymph.	75.0 (reference: 12.0-35.5%)
CD4/CD8 Ratio	0.10 (reference: 0.92-3.72)

A chest X-ray was done on admission which was unremarkable and showed no radiographic evidence of any acute cardiopulmonary disease. 

The diagnosis of VZV reactivation during this event was made clinically based on the characteristics of the rash, the patient’s typical signs and symptoms, the context of being immunosuppressed in view of his untreated HIV and his history of a previous VZV episode. The patient was admitted to the intensive care unit (ICU) in view of his potential to withdraw from alcohol and was started on acyclovir for the VZV and cefazolin to cover for a possible superimposed cellulitis following the recommendations of the Infectious Disease team. He was also started on gabapentin, which over the next 48 hours led to improvement in the patient's neuropathic pain. The next day, the patient was started on Biktarvy (bictegravir/emtricitabine/tenofovir) and was counseled on the importance of compliance with his HIV medications. He remained in the hospital for 5 days until his rash and neurologic pain had improved. On discharge, the long-term plan discussed with the patient was to refer him to our outpatient HIV clinic once his pain and rash had improved and he was no longer transmissible. 

## Discussion

There is an overall consensus that the Pfizer-BioNTech COVID-19 vaccine has relatively minor side effects in a majority of people, and is considered safe. Nevertheless, there have been side effects that have been reported in the literature. Side effects range from localized pain, tenderness, redness at the vaccination site, fever, chills, fatigue, muscle aches, there have been reported cases of dermatologic side such as VZV previously discussed in the case above. There are previously documented cases of Herpes zoster reactivation during the acute phase of COVID 19 infections in both the younger and older population [5]. As per the United States Vaccine Adverse Event Report System, there were 2512 VZV cases (totaling roughly 1.3% of total reported events) after patients received the COVID Pfizer vaccine, 1763 (0.9%) after receiving COVID Moderna vaccine, and 302 (0.7%) after Janssen vaccine [6].

There was another analysis done by Fathy et.al where they analyzed the first 35 cases of VZV reactivation reported in the International League of Dermatologic Societies’ COVID-19 Dermatology Registry by April 2021 [7]. Out of the 35 cases, 19 had been secondary to the Pfizer vaccine and 16 due to the Moderna vaccine [7]. The median age of the 35 cases was 46 years old and 77% of them had a reaction after the first dose of the vaccine compared to only 23% that had a reaction after the second dose [7]. Moreover, only 2.9% of the patients had the rash on the non-vaccinated arm and the majority including 46% had the rash on their back [7]. Finally, only 1 of the 35 patients was immunocompromised as a comorbidity [7]. Unfortunately, a limitation of this study was that the baseline characteristics were not divided into the Moderna versus Pfizer recipients.

While we could not 100% conclude that the Pfizer COVID-19 vaccine was the cause of the shingles flare in view of the patient’s untreated HIV infection, we could definitely attribute the flare to the vaccine in view of the several case reports and case series of shingles post-vaccination reported in the literature in addition to the temporal relationship of the events. The rare occurrence of such severe shingles occurring in a young patient could definitely be explained by his AIDS-related immunocompromised state and his CD4 < 200.

## Conclusions

In view of very little information in the literature elaborating on the mechanism behind the increased incidence of shingles following the COVID vaccine, more studies will be needed to clarify the exact mechanism of vaccine-induced reactivation of VZV. Finally, in regards to the very painful outcome of shingles, an early clinical diagnosis and differential of shingles are essential to prevent morbidity in patients. 
